# A Risk Scoring System Integrating Clinical and Ultrasonographic Parameters for Predicting Hypothyroidism After Hemithyroidectomy

**DOI:** 10.3390/diagnostics16162550

**Published:** 2026-08-13

**Authors:** Yusuf Öztürk, Hatice Erdoğan Özbuğday, Zuhal Dağ, Muhammet Kocabaş

**Affiliations:** 1 Department of Endocrinology and Metabolism, Sakarya University Training and Research Hospital, Adapazarı 54290, Türkiye; zuhaldag02@gmail.com; 2 Department of Endocrinology and Metabolism, Necmettin Erbakan University Faculty of Medicine, Konya 42090, Türkiye; hatice.erdogan@erbakan.edu.tr (H.E.Ö.); mkocabas@erbakan.edu.tr (M.K.)

**Keywords:** hemithyroidectomy, postoperative hypothyroidism, remnant thyroid volume, thyroid parenchymal heterogeneity, thyroid-stimulating hormone

## Abstract

**Background/Objectives**: Postoperative hypothyroidism is a common complication following hemithyroidectomy and may necessitate lifelong levothyroxine replacement therapy. Although several clinical predictors have been identified, clinically applicable risk models integrating both clinical and ultrasonographic parameters remain limited. This study aimed to identify independent predictors of postoperative hypothyroidism and to develop practical risk scoring systems with and without ultrasonographic parameters based on these predictors. **Methods**: This observational cohort study included 95 patients who underwent hemithyroidectomy and were followed for at least 6 months. Clinical characteristics, thyroid autoantibodies, and ultrasonographic features of the remnant thyroid lobe were evaluated. Independent predictors of postoperative hypothyroidism were identified using multivariable logistic regression analysis, and two simplified risk scoring systems were developed based on the regression coefficients. **Results**: Postoperative hypothyroidism developed in 39 of the 95 patients (41.1%). In the model incorporating ultrasonographic parameters, preoperative TSH, a low remnant thyroid volume-to-body surface area ratio, and heterogeneous remnant thyroid parenchyma were independent predictors of postoperative hypothyroidism. The corresponding score stratified patients into low-, intermediate-, and high-risk categories with observed hypothyroidism rates of 9.1%, 44.7%, and 100.0%, respectively, and showed an AUC of 0.858. In the model without ultrasonographic parameters, preoperative TSH and thyroid autoantibody positivity were independent predictors; the corresponding score showed an AUC of 0.745. **Conclusions**: The proposed risk scoring systems may serve as practical tools for identifying patients at increased risk of postoperative hypothyroidism following hemithyroidectomy according to the availability of ultrasonographic assessment. External validation in independent cohorts is warranted before routine clinical implementation.

## 1. Introduction

Hemithyroidectomy is a widely accepted surgical procedure for the management of unilateral benign thyroid nodules, selected indeterminate thyroid nodules, and appropriately selected patients with low-risk differentiated thyroid carcinoma [1]. Compared with total thyroidectomy, it preserves functional thyroid tissue, is associated with lower rates of permanent hypoparathyroidism and recurrent laryngeal nerve injury, and does not invariably require lifelong levothyroxine replacement therapy. Accordingly, current clinical guidelines recommend hemithyroidectomy as the preferred surgical approach for appropriately selected patients [1]. Nevertheless, the functional capacity of the remaining thyroid lobe varies considerably among individuals, and a substantial proportion of patients subsequently develop postoperative hypothyroidism during follow-up [2,3].

Postoperative hypothyroidism is one of the most common long-term complications following hemithyroidectomy, with reported incidence rates varying widely across studies [4,5,6,7]. This variability is largely attributable to differences in patient selection, duration of follow-up, definitions of hypothyroidism, and thresholds for initiating levothyroxine therapy [8,9,10]. Importantly, the clinical significance of postoperative hypothyroidism extends beyond biochemical abnormalities alone. Although levothyroxine replacement therapy is generally safe, effective, and inexpensive, lifelong treatment requires continuous medication use, regular biochemical monitoring, dose adjustments, and long-term healthcare utilization, resulting in a cumulative burden for both patients and healthcare systems [11]. Moreover, despite achieving biochemical euthyroidism, a subset of patients receiving levothyroxine continue to experience persistent symptoms and impaired quality of life. This clinical entity has recently been referred to as syndrome T (thyroprivic syndrome) in Europe and SORSHOT (syndrome of residual symptoms of hypothyroidism on T4) in the United States [12,13]. Therefore, accurately identifying patients who are likely to maintain euthyroidism after hemithyroidectomy may help avoid unnecessary lifelong thyroid hormone replacement, reduce long-term treatment burden, improve patient counseling, and optimize individualized postoperative surveillance.

Numerous studies have investigated potential predictors of hypothyroidism following hemithyroidectomy. Elevated preoperative thyroid-stimulating hormone (TSH) levels, thyroid autoimmunity, Hashimoto thyroiditis, residual thyroid volume, and various histopathological characteristics have all been associated with an increased risk of postoperative hypothyroidism [3,6,14,15,16,17]. However, the reported independent predictors have not been entirely consistent across studies. Furthermore, most published prediction models rely on a limited number of variables or do not provide a practical risk stratification tool that can be readily incorporated into routine clinical practice [2,14]. Consequently, there remains a need for a simple, reliable, and clinically applicable model capable of accurately predicting the risk of postoperative hypothyroidism after hemithyroidectomy.

We hypothesized that integrating preoperative biochemical markers with ultrasonographic characteristics of the remnant thyroid lobe assessed using a standardized postoperative ultrasonographic examination would improve prediction of postoperative hypothyroidism. Therefore, this study aimed to identify independent predictors of hypothyroidism occurring within the first six months after hemithyroidectomy, compare different prediction models, and develop a simple risk scoring system that can be readily applied in routine clinical practice.

## 2. Materials and Methods

### 2.1. Study Design and Patient Selection

This observational cohort study was conducted at the Division of Endocrinology and Metabolism, Necmettin Erbakan University Meram Faculty of Medicine, and the Department of Endocrinology and Metabolism, Sakarya Training and Research Hospital. The study protocol was approved by the Non-Drug and Non-Medical Device Research Ethics Committee of Necmettin Erbakan University (Approval No. 2026/6263; 9 January 2026) and was conducted in accordance with the principles of the Declaration of Helsinki.

Consecutive adult patients referred to the endocrinology outpatient clinic for routine postoperative follow-up after hemithyroidectomy were screened for eligibility. Hemithyroidectomy was defined as surgical removal of one thyroid lobe together with the isthmus while preserving the contralateral thyroid lobe.

Patients were eligible if they were aged ≥18 years, had normal preoperative TSH and free thyroxine (fT4) levels before surgery, underwent their first postoperative endocrinology evaluation approximately 4–6 weeks after surgery, and had at least 6 months of thyroid function follow-up. Accordingly, all patients included in the study were euthyroid before surgery and were not receiving thyroid hormone replacement therapy at the time of hemithyroidectomy.

**Figure 1:**
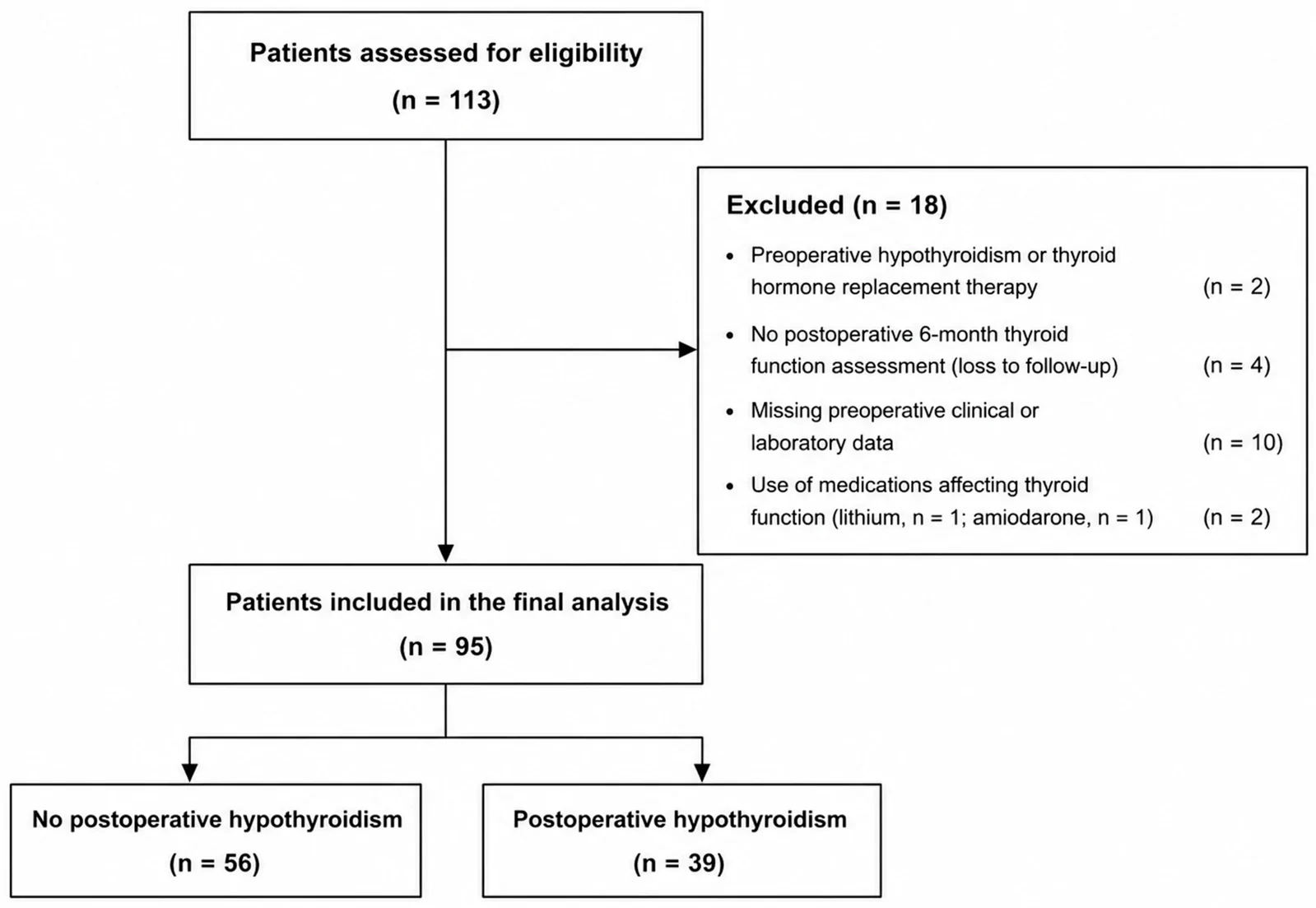
Flow diagram of patient selection and study cohort formation.

Patients were excluded if they had preoperative hypothyroidism or were receiving levothyroxine therapy, had overt or subclinical hyperthyroidism, including toxic thyroid nodules, had undergone previous thyroid surgery, were taking medications known to affect thyroid function (e.g., lithium or amiodarone), had incomplete preoperative clinical or laboratory data, or lacked at least 6 months of postoperative follow-up. Thyroid scintigraphy was not routinely performed as part of the study protocol and was performed only when clinically indicated during the preoperative evaluation. The patient selection process is illustrated in Figure 1.

### 2.2. Clinical, Laboratory, Ultrasonographic, and Histopathological Assessment

Baseline demographic data, including age, sex, height, body weight, body mass index (BMI), and the operated thyroid lobe, were obtained from the electronic medical records. Preoperative fine-needle aspiration (FNA) cytology results of the index thyroid nodule were recorded according to the Bethesda System for Reporting Thyroid Cytopathology. Preoperative serum TSH, fT4, anti-thyroid peroxidase antibody (anti-TPO), and anti-thyroglobulin antibody (anti-Tg) levels were also collected. Serum free triiodothyronine (fT3) and TSH receptor antibody (TRAb) measurements were not routinely available for all patients and were therefore not included in the statistical analyses. Using the preoperative TSH and fT4 measurements, Jostel’s TSH Index (TSHI), an indicator of pituitary thyrotropic function, and the thyroid secretory capacity (SPINA-GT), an indicator of the maximum secretory capacity of the thyroid gland, were calculated using validated equations [18,19].

As part of the routine postoperative follow-up protocol, all patients were evaluated in the endocrinology outpatient clinic approximately 4–6 weeks after hemithyroidectomy. During this visit, serum TSH and fT4 levels were remeasured, and ultrasonographic examination of the remnant thyroid lobe was performed. Patients subsequently underwent routine follow-up evaluations at 3 and 6 months after surgery, including thyroid function testing. For the assessment of postoperative thyroid function, the peak TSH and lowest fT4 concentrations recorded during the 6-month follow-up were used. In patients who initiated levothyroxine replacement therapy during follow-up, only thyroid function measurements obtained before treatment initiation were considered for these parameters, thereby ensuring that the reported values reflected native postoperative thyroid function.

Ultrasonographic examinations were performed using a standardized imaging protocol. At Necmettin Erbakan University Meram Faculty of Medicine, examinations were performed using an Acuson Juniper Ultrasound System (Siemens Healthineers, Berlin, Germany) equipped with a 12L3 linear transducer (3.6–12.9 MHz). At Sakarya Training and Research Hospital, examinations were performed using a LOGIQ ultrasound system (GE HealthCare, Chicago, IL, USA) with a linear-array transducer. At both centers, all ultrasonographic examinations were performed jointly by two endocrinologists experienced in thyroid ultrasonography, and all measurements and imaging findings were recorded by consensus. The examiners were blinded to the postoperative thyroid function test results.

The craniocaudal length, mediolateral width, and anteroposterior depth of the remnant thyroid lobe were measured. Remnant thyroid volume was calculated according to the method described by Brunn et al. using the formula: length × width × depth × 0.479 [20]. To account for interindividual differences in body size, body surface area (BSA) was calculated using the Mosteller formula (BSA [m^2^] = √[(height [cm] × weight [kg])/3600]) [21]. The remnant thyroid volume-to-BSA ratio (mL/m^2^) was subsequently calculated by dividing the remnant thyroid volume by the patient’s BSA.

Thyroid parenchymal echotexture was classified as homogeneous or heterogeneous based on the consensus assessment of the two experienced endocrinologists. Heterogeneous parenchyma was defined as the presence of diffuse and irregular alterations in echogenicity compared with normal thyroid tissue, including coarse parenchymal echotexture, patchy hypoechoic areas, and/or a pseudonodular appearance. This assessment was based on the overall ultrasonographic appearance of the thyroid parenchyma using morphological criteria routinely applied in clinical practice [22,23].

Final histopathological diagnoses were obtained from routine pathology reports. Patients were classified as having benign or malignant pathology according to the final histopathological diagnosis. Histologically malignant thyroid neoplasms were classified within the malignant group, whereas benign thyroid lesions, follicular adenoma, chronic lymphocytic thyroiditis, and noninvasive follicular thyroid neoplasm with papillary-like nuclear features (NIFTP) were classified within the benign group.

### 2.3. Postoperative Follow-Up and Outcome Definition

Patients were routinely followed in the endocrinology outpatient clinic at approximately 4–6 weeks, 3 months, and 6 months after hemithyroidectomy. At each follow-up visit, a clinical assessment was performed, and serum TSH and fT4 levels were measured. Additional laboratory tests were obtained when clinically indicated. The laboratory reference ranges were 0.27–4.20 μIU/mL for TSH, 0.92–1.70 ng/dL for fT4, 0–9 IU/mL for anti-TPO, and 0–115 IU/mL for anti-Tg.

The primary outcome was the development of postoperative hypothyroidism within the first 6 months after hemithyroidectomy. Subclinical hypothyroidism was defined as an elevated serum TSH level above the upper limit of the laboratory reference range with a normal fT4 level, whereas overt hypothyroidism was defined as an elevated serum TSH level accompanied by a decreased fT4 level.

Because thyroid dysfunction occurring early after hemithyroidectomy may be transient, levothyroxine replacement was not routinely initiated solely on the basis of an isolated early postoperative elevation in TSH. Thyroid function was reassessed during follow-up, and the decision to initiate levothyroxine was based on the persistence and severity of biochemical hypothyroidism together with clinical assessment, in accordance with current international guideline recommendations [24,25]. Patients who developed postoperative hypothyroidism during the 6-month follow-up and required levothyroxine replacement were classified into the hypothyroidism group, whereas patients who maintained or recovered euthyroidism without levothyroxine replacement were classified into the euthyroidism group.

### 2.4. Statistical Analysis

Statistical analyses were performed using IBM SPSS Statistics for Windows, Version 22.0 (IBM Corp., Armonk, NY, USA). The normality of continuous variables was assessed using the Shapiro–Wilk test. Normally distributed variables are presented as mean ± standard deviation (SD), whereas non-normally distributed variables are presented as median (interquartile range [IQR]). Categorical variables are expressed as numbers and percentages.

Comparisons between the hypothyroidism and euthyroidism groups were performed using Student’s *t*-test for normally distributed continuous variables, the Mann–Whitney U test for non-normally distributed variables, and the chi-square test or Fisher’s exact test, as appropriate, for categorical variables.

Receiver operating characteristic (ROC) curve analysis was performed to evaluate the discriminative ability of preoperative TSH, TSHI, SPINA-GT, remnant thyroid volume, and the remnant thyroid volume-to-BSA ratio for predicting postoperative hypothyroidism. The area under the ROC curve (AUC) with its 95% confidence interval (CI) was calculated for each variable. Optimal cutoff values were determined using the Youden index and subsequently used to dichotomize continuous predictors for logistic regression analyses.

To identify factors associated with postoperative hypothyroidism, univariable binary logistic regression analyses were initially performed. Variables with a *p* value < 0.10 in the univariable analyses, together with variables considered clinically relevant, were considered for multivariable analysis. TSHI and SPINA-GT were included in the univariable analyses but were excluded from the multivariable models because they are calculated parameters derived from biochemical variables included among the candidate predictors, thereby avoiding potential spurious associations and multicollinearity. Multicollinearity among variables included in the multivariable models was assessed using variance inflation factors (VIFs). Results are presented as odds ratios (ORs) with 95% confidence intervals (CIs) and corresponding *p* values. Model calibration was assessed using the Hosmer–Lemeshow goodness-of-fit test, and explanatory performance was evaluated using the Nagelkerke R^2^ statistic.

To facilitate clinical application, two simplified risk scoring systems were developed based on the β coefficients of the independent predictors identified in the two multivariable models: Model 1, which combined biochemical and ultrasonographic parameters, and Model 2, which excluded ultrasonographic parameters. Continuous variables were dichotomized according to the optimal cutoff values determined by ROC analysis. For each model, weighted points were assigned according to the relative magnitude of the corresponding β coefficients and rounded to obtain simple integer scores while preserving their relative contributions. Total scores were calculated separately for each model, and patients were subsequently categorized into low-, intermediate-, and high-risk groups according to the observed incidence of postoperative hypothyroidism across score levels. The discriminative performance of each scoring system was evaluated using ROC curve analysis.

All statistical tests were two-sided, and a *p* value < 0.05 was considered statistically significant.

## 3. Results

### 3.1. Study Population Characteristics

A total of 95 euthyroid patients who met the predefined eligibility criteria were included in the study. During the 6-month follow-up period, postoperative hypothyroidism developed in 39 patients (41.1%), whereas 56 patients (58.9%) remained euthyroid. The demographic, clinical, laboratory, ultrasonographic, and histopathological characteristics of the study population are summarized in Table 1.

No significant differences were observed between the hypothyroidism and euthyroidism groups regarding age, sex, BMI, preoperative fT4 levels, Bethesda cytological category, malignancy rate, or the side of hemithyroidectomy (all *p* > 0.05).

In contrast, patients who developed postoperative hypothyroidism had significantly higher preoperative TSH levels (*p* < 0.001). Preoperative TSHI was also significantly higher, whereas SPINA-GT was significantly lower, in patients who subsequently developed postoperative hypothyroidism (both *p* < 0.001). Anti-TPO positivity (*p* = 0.003), anti-Tg positivity (*p* = 0.011), and the presence of thyroid autoantibodies (*p* = 0.028) were also significantly more frequent in the hypothyroidism group.

Ultrasonographic assessment demonstrated that both the remnant thyroid volume and the remnant thyroid volume-to-BSA ratio were significantly lower in patients who developed postoperative hypothyroidism (both *p* ≤ 0.001). In addition, heterogeneous remnant thyroid parenchyma was significantly more common in the hypothyroidism group (*p* < 0.001). Histopathological thyroiditis was also observed more frequently among patients who developed postoperative hypothyroidism (*p* < 0.001) (Table 1).

### 3.2. Univariable Predictors of Postoperative Hypothyroidism

**Figure 2:**
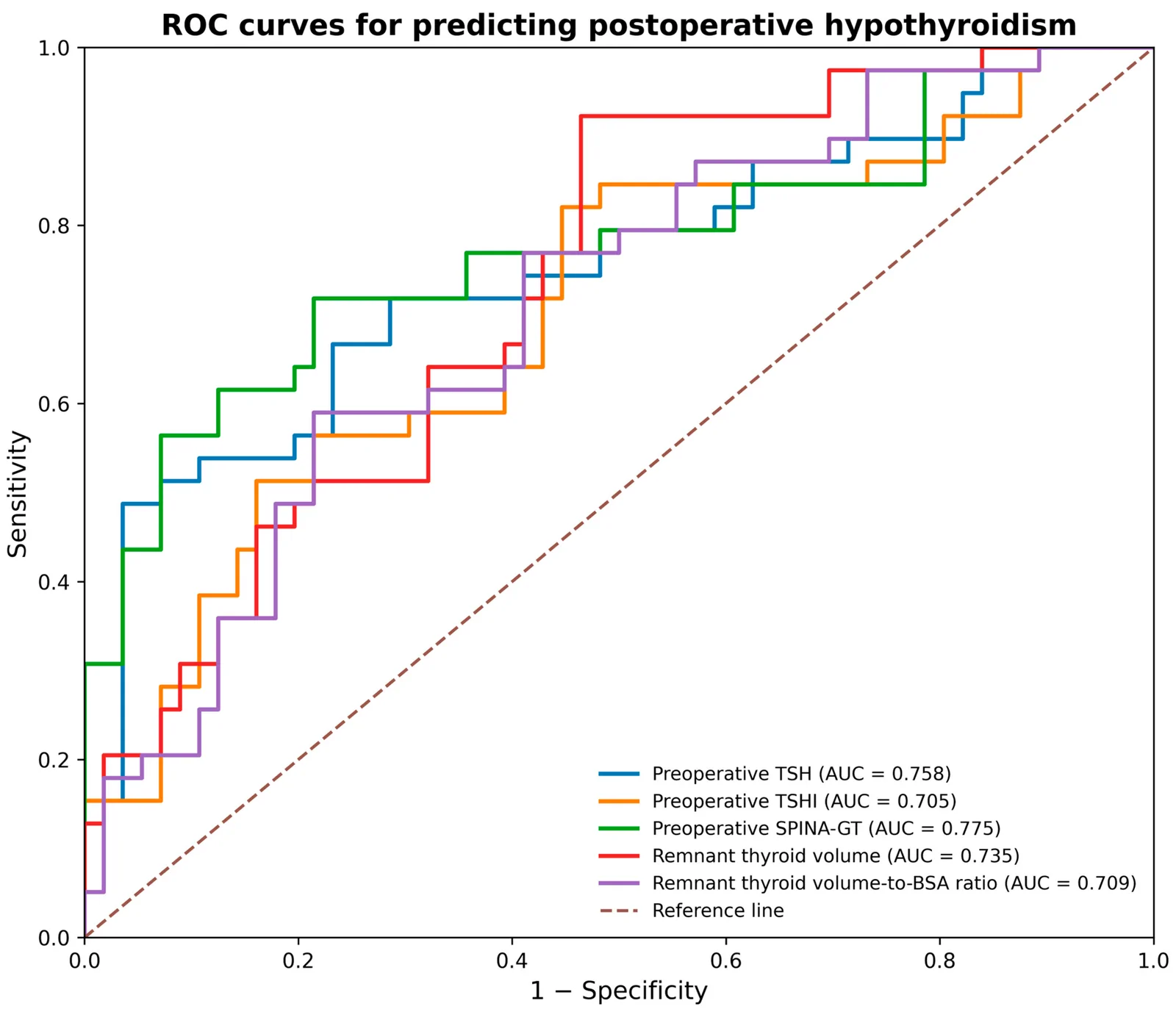
Receiver operating characteristic (ROC) curves of preoperative TSH, TSHI, SPINA-GT, remnant thyroid volume, and remnant thyroid volume-to-BSA ratio for predicting postoperative hypothyroidism after hemithyroidectomy.

The discriminative performance of preoperative TSH, TSHI, SPINA-GT, remnant thyroid volume, and the remnant thyroid volume-to-BSA ratio for predicting postoperative hypothyroidism was evaluated using ROC curve analysis (Figure 2). The optimal cutoff values were ≥1.77 mIU/L for preoperative TSH (AUC = 0.758), ≥2.49 for TSHI (AUC = 0.705), ≤2.10 pmol/s for SPINA-GT (AUC = 0.775), ≤4.24 mL for remnant thyroid volume (AUC = 0.735), and ≤1.84 mL/m^2^ for the remnant thyroid volume-to-BSA ratio (AUC = 0.709).

In the univariable logistic regression analysis, preoperative TSH ≥ 1.77 mIU/L (OR, 5.63; 95% CI, 2.30–13.75; *p* < 0.001), TSHI ≥ 2.49 (OR, 2.76; 95% CI, 1.18–6.43; *p* = 0.019), SPINA-GT ≤ 2.10 pmol/s (OR, 6.36; 95% CI, 2.57–15.76; *p* < 0.001), remnant thyroid volume ≤ 4.24 mL (OR, 3.77; 95% CI, 1.59–8.92; *p* = 0.003), remnant thyroid volume-to-BSA ratio ≤ 1.84 mL/m^2^ (OR, 5.27; 95% CI, 2.14–13.00; *p* < 0.001), anti-TPO positivity (OR, 5.10; 95% CI, 1.64–15.86; *p* = 0.005), anti-Tg positivity (OR, 5.30; 95% CI, 1.33–21.09; *p* = 0.018), the presence of at least one positive thyroid autoantibody (OR, 3.00; 95% CI, 1.10–8.17; *p* = 0.032), and heterogeneous remnant thyroid parenchyma (OR, 13.65; 95% CI, 3.63–51.32; *p* < 0.001) were significantly associated with postoperative hypothyroidism (Table 2).

### 3.3. Multivariable Prediction Models for Postoperative Hypothyroidism

Two multivariable logistic regression models were constructed to evaluate predictors of postoperative hypothyroidism, one including ultrasonographic parameters and the other excluding ultrasonographic parameters (Table 3). In Model 1, which included ultrasonographic parameters, preoperative TSH ≥ 1.77 mIU/L (OR, 5.06; 95% CI, 1.69–15.21; *p* = 0.004), a remnant thyroid volume-to-BSA ratio ≤ 1.84 mL/m^2^ (OR, 6.62; 95% CI, 2.14–20.52; *p* = 0.001), and heterogeneous remnant thyroid parenchyma (OR, 20.83; 95% CI, 4.49–96.70; *p* < 0.001) were independently associated with postoperative hypothyroidism. The model χ^2^ was 46.51 (*p* < 0.001), the Nagelkerke R^2^ was 0.522, the Hosmer–Lemeshow test *p* value was 0.768, and the overall classification accuracy was 80.0%. In Model 2, which excluded ultrasonographic parameters, preoperative TSH ≥ 1.77 mIU/L (OR, 6.14; 95% CI, 2.41–15.67; *p* < 0.001) and the presence of at least one positive thyroid autoantibody (OR, 3.56; 95% CI, 1.17–10.85; *p* = 0.026) were independently associated with postoperative hypothyroidism. The model χ^2^ was 20.93 (*p* < 0.001), the Nagelkerke R^2^ was 0.267, the Hosmer–Lemeshow test *p* value was 0.124, and the overall classification accuracy was 70.5%. VIF values ranged from 1.001 to 1.078 across both models (Table 3).

### 3.4. Development of the Simplified Clinical Risk Score

Two simplified clinical risk scoring systems were developed based on the predictors included in the corresponding multivariable models (Table 4).

For Model 1, which incorporated both biochemical and ultrasonographic parameters, 1 point was assigned for preoperative TSH ≥ 1.77 mIU/L, 1 point for a remnant thyroid volume-to-BSA ratio ≤ 1.84 mL/m^2^, and 2 points for heterogeneous remnant thyroid parenchyma, based on the relative magnitude of the corresponding β coefficients. The resulting score ranged from 0 to 4 points. Patients were classified as low risk (0 points), intermediate risk (1–2 points), and high risk (3–4 points), with observed rates of postoperative hypothyroidism of 9.1%, 44.7%, and 100.0%, respectively. The AUC for Model 1 was 0.858 (95% CI, 0.779–0.936). At a cutoff of ≥2 points, sensitivity was 71.8% and specificity was 85.7%.

For Model 2, which excluded ultrasonographic parameters, 2 points were assigned for preoperative TSH ≥ 1.77 mIU/L and 1 point for the presence of at least one positive thyroid autoantibody, yielding a total score ranging from 0 to 3 points. Patients were classified as low risk (0–1 points), intermediate risk (2 points), and high risk (3 points), with observed rates of postoperative hypothyroidism of 23.1%, 51.5%, and 100.0%, respectively. The AUC for Model 2 was 0.745 (95% CI, 0.642–0.849). At a cutoff of ≥2 points, sensitivity was 69.2% and specificity was 71.4% (Table 4).

## 4. Discussion

One of the principal advantages of hemithyroidectomy is the preservation of functioning thyroid tissue, which may eliminate the need for lifelong levothyroxine replacement therapy in appropriately selected patients. However, this benefit depends on the ability of the remnant thyroid tissue to maintain adequate hormonal function after surgery. Therefore, identifying reliable predictors of postoperative hypothyroidism and developing clinically applicable prediction models remain important for preoperative risk stratification and postoperative management. Preoperative TSH, a low remnant thyroid volume-to-BSA ratio, and heterogeneous remnant thyroid parenchyma were identified as independent predictors of postoperative hypothyroidism in the model incorporating ultrasonographic parameters. Based on the two multivariable prediction models, we developed two simplified clinical risk scores: one combining biochemical and ultrasonographic parameters and another based on biochemical parameters without ultrasonographic assessment. Both scoring systems stratified patients into low-, intermediate-, and high-risk categories, with increasing observed rates of postoperative hypothyroidism across risk categories.

A recent systematic review and meta-analysis by Cooper et al. identified elevated preoperative TSH levels, Hashimoto thyroiditis, and thyroid autoantibody positivity as the most consistent risk factors for hypothyroidism after hemithyroidectomy [2]. Consistent with these findings, higher preoperative TSH levels and markers suggestive of thyroid autoimmunity were associated with postoperative hypothyroidism in our cohort. In contrast, other factors reported in the meta-analysis, including older age, female sex, and right-sided hemithyroidectomy, were not significantly associated with postoperative hypothyroidism in our study. Although the optimal preoperative TSH cutoff values reported across studies show modest variation, they consistently remain well below the upper limit of the laboratory reference range. Cao et al. reported an optimal cutoff value of 2.17 mIU/L [14], Tahab et al. reported 2.61 mIU/L [26], and Arraf et al. reported 2.0 mIU/L [3]. In our study, ROC curve analysis identified an optimal cutoff value of 1.77 mIU/L, which is likewise substantially below the upper reference limit used in routine clinical practice. Differences among studies may reflect variations in patient characteristics, the definition of postoperative hypothyroidism, follow-up duration, statistical methodology, and selection bias. Nevertheless, the consistent observation across studies suggests that even preoperative TSH values within the conventional reference range may reflect a reduced functional thyroid reserve, thereby increasing susceptibility to postoperative hypothyroidism. This interpretation is further supported by our analysis of calculated thyroid homeostasis parameters. Patients who subsequently developed postoperative hypothyroidism had higher preoperative TSHI and lower SPINA-GT, indicating alterations in indices reflecting pituitary thyrotropic function and, importantly, reduced thyroid secretory capacity before surgery. Taken together with the higher preoperative TSH levels, these findings support the presence of a lower functional thyroid reserve before hemithyroidectomy in patients who subsequently develop hypothyroidism. Importantly, the principal contribution of our study is not the identification of another preoperative TSH cutoff value. Although previous studies have consistently demonstrated that preoperative TSH is a robust biochemical predictor, our findings indicate that TSH alone does not capture the full risk of postoperative hypothyroidism. This was evident in both of the newly developed scoring systems, in which additional information beyond preoperative TSH was required for risk stratification. In the model incorporating ultrasonographic parameters, preoperative TSH was complemented by the remnant thyroid volume-to-BSA ratio and parenchymal heterogeneity, thereby integrating biochemical function with the amount and structural characteristics of the remaining thyroid tissue. In the model without ultrasonographic parameters, thyroid autoantibody status provided additional information beyond preoperative TSH and allowed risk stratification when ultrasonographic assessment was not incorporated. Thus, rather than relying on TSH as an isolated predictor, the two scoring systems provide complementary approaches to risk assessment according to the availability of ultrasonographic information.

Thyroid autoimmunity, including Hashimoto thyroiditis and thyroid autoantibody positivity, is widely recognized as one of the most consistent risk factors for the development of postoperative hypothyroidism after hemithyroidectomy [2,6,15,16]. These findings support the concept that chronic autoimmune injury reduces the functional reserve of the remnant thyroid tissue, thereby increasing susceptibility to postoperative hypothyroidism. Consistent with the existing literature, thyroid autoantibody positivity was significantly associated with postoperative hypothyroidism in our univariable analysis. An important finding of our study, however, is that ultrasonographic parenchymal heterogeneity appeared to provide greater predictive value than serological markers of autoimmunity. In Model 1, which incorporated preoperative TSH together with ultrasonographic parameters, preoperative TSH, the remnant thyroid volume-to-BSA ratio, and heterogeneous remnant thyroid parenchyma were independently associated with postoperative hypothyroidism. This model showed a Nagelkerke R^2^ of 0.522 and an overall classification accuracy of 80.0%. In Model 2, which was constructed without ultrasonographic parameters, preoperative TSH and the presence of ≥1 positive thyroid autoantibody independently predicted postoperative hypothyroidism; however, this model showed a lower Nagelkerke R^2^ of 0.267 and an overall classification accuracy of 70.5%. One possible explanation for this difference is that thyroid autoantibody positivity indicates the presence of thyroid autoimmunity but does not necessarily imply that substantial structural damage to the thyroid tissue has already occurred. Structural alterations associated with autoimmune thyroiditis may evolve over the course of the disease, and thyroid atrophy has been suggested to represent a relatively late phenomenon in its progression [27]. In contrast, ultrasonographic parenchymal heterogeneity may more directly reflect inflammatory and destructive changes within the thyroid tissue [28]. Therefore, parenchymal heterogeneity may provide information complementary to thyroid autoantibody status and may more closely reflect the functional reserve of the remnant thyroid tissue. Nevertheless, this interpretation should not be taken to diminish the clinical importance of thyroid autoantibodies. Moreover, because our study evaluated only the first six postoperative months, the prognostic significance of thyroid autoantibodies beyond the early postoperative period may have been underestimated. Future studies with longer follow-up are needed to clarify the relative contributions of serological and ultrasonographic markers over time. From a clinical perspective, our findings suggest two complementary approaches. In centers where thyroid ultrasonography is routinely performed by experienced operators, assessment of remnant thyroid parenchymal heterogeneity together with remnant thyroid volume relative to body size may substantially improve postoperative risk stratification. Conversely, in settings where high-quality ultrasonographic evaluation is not readily available, combining preoperative TSH with thyroid autoantibody status may still provide a practical and clinically useful alternative for identifying patients at increased risk of postoperative hypothyroidism, although with lower predictive performance than the ultrasonography-based approach.

The role of remnant thyroid volume in the development of postoperative hypothyroidism remains controversial. Cherni et al. [29] reported that a remnant thyroid volume < 3.57 cm^3^ was significantly associated with postoperative hypothyroidism, whereas Amitani et al. [30] identified a low remnant thyroid index (RTI) as an independent risk factor. In contrast, Arraf et al. [3] found no significant association between remnant thyroid volume and postoperative hypothyroidism and suggested that biochemical markers may be more informative than anatomical measurements. In our study, both remnant thyroid volume and the remnant thyroid volume-to-BSA ratio were significantly associated with postoperative hypothyroidism, whereas only the remnant thyroid volume-to-BSA ratio remained an independent predictor in the multivariable analysis. Nevertheless, our findings also indicate that remnant thyroid volume alone is insufficient for identifying patients at high risk. Within the simplified risk score derived from Model 1, the remnant thyroid volume-to-BSA ratio contributed the same number of points as preoperative TSH but did not classify patients as high risk in isolation. Instead, its predictive value became clinically more meaningful when interpreted together with preoperative TSH and ultrasonographic evidence of heterogeneous remnant thyroid parenchyma. These findings suggest that remnant thyroid volume provides complementary rather than standalone prognostic information and that its clinical utility may be enhanced when interpreted alongside biochemical and structural markers reflecting thyroid functional reserve.

One of the major strengths of our study is the translation of the identified independent predictors into two simplified clinical risk scoring systems that can be applied according to the availability of ultrasonographic assessment. Although numerous clinical, biochemical, and ultrasonographic risk factors for hypothyroidism after hemithyroidectomy have been reported, studies integrating these variables into simple and practical risk prediction tools remain limited. The Model 1 score combines preoperative TSH, the remnant thyroid volume-to-BSA ratio, and heterogeneous remnant thyroid parenchyma, whereas the Model 2 score, designed for settings where ultrasonographic parameters are unavailable, combines preoperative TSH with thyroid autoantibody status. Both scoring systems stratified patients into low-, intermediate-, and high-risk categories for postoperative hypothyroidism, although the Model 1 score demonstrated greater discriminative ability than the Model 2 score (AUC, 0.858 vs. 0.745). By providing complementary models with and without ultrasonographic parameters, this approach may facilitate individualized postoperative risk assessment across different clinical settings. Another clinically relevant aspect of our findings is that the Model 1 scoring system may also have potential for preoperative risk assessment. In the present study, remnant thyroid volume was measured 4–6 weeks after surgery. As substantial compensatory hypertrophy of the remaining thyroid tissue is unlikely to have fully developed during this early postoperative period, the measured remnant volume may reasonably reflect the immediate postoperative thyroid remnant. Because remnant thyroid volume can also be estimated preoperatively by ultrasonography, the Model 1 scoring system may have potential for preoperative risk stratification. If validated in prospective studies, such an approach could facilitate preoperative patient counseling, individualized postoperative follow-up, and closer thyroid function monitoring in patients at increased risk of postoperative hypothyroidism. However, this potential application was not directly evaluated in the present study and should therefore be confirmed in future prospective validation studies.

This study has several limitations. First, although the study was conducted at two tertiary referral centers, the generalizability of the findings to other institutions and patient populations may be limited. Second, the relatively modest sample size may have reduced the ability to detect variables with smaller effect sizes and limited more detailed subgroup analyses. Third, the follow-up period was limited to six months; therefore, late-onset hypothyroidism, compensatory changes in the remnant thyroid tissue over time, and the long-term impact of thyroid autoimmunity could not be fully evaluated. In addition, although ultrasonographic assessments were performed according to a standardized protocol by two experienced endocrinologists through consensus review, thyroid ultrasonography is inherently operator-dependent, and some degree of measurement variability cannot be completely excluded. Finally, the prediction models and their corresponding simplified clinical risk scores were developed and internally evaluated within the same patient cohort without external validation. Therefore, prospective validation in larger, independent, and multicenter cohorts is warranted before these models and scoring systems can be widely implemented in routine clinical practice.

## 5. Conclusions

In conclusion, our study demonstrates that integrating preoperative TSH with ultrasonographic assessment of the remnant thyroid improves the prediction of hypothyroidism after hemithyroidectomy. Among the evaluated variables, heterogeneous remnant thyroid parenchyma emerged as one of the strongest independent predictors of postoperative hypothyroidism. When ultrasonography is available, combining preoperative TSH with remnant thyroid volume-to-BSA ratio and parenchymal heterogeneity may provide a more comprehensive assessment of postoperative hypothyroidism risk. In settings where ultrasonographic evaluation is not feasible, a model based on biochemical variables alone may represent a practical alternative for postoperative risk stratification. Prospective external validation is warranted before these models are implemented in routine clinical practice.

## Figures and Tables

**Table 1:** Baseline demographic, laboratory, ultrasonographic, and histopathological characteristics of the study population according to postoperative thyroid function.

Variable	Overall (*n* = 95)	Euthyroidism (*n* = 56)	Hypothyroidism (*n* = 39)	*p* Value
Demographic characteristics				
Age, years	44 (36–57)	42.5 (36–58)	48 (36–57)	0.591
Female sex, *n* (%)	84 (88.4)	47 (83.9)	37 (94.9)	0.101
BMI, kg/m^2^	30.0 (27.1–33.3)	30.3 (27.7–34.6)	30.0 (27.0–32.6)	0.151
Preoperative laboratory findings				
TSH, mIU/L	1.70 (1.32–2.40)	1.50 (1.12–1.90)	2.50 (1.61–3.30)	**<0.001**
fT4, ng/dL	1.09 (1.00–1.20)	1.10 (1.00–1.25)	1.07 (1.00–1.14)	0.351
TSHI	2.48 (2.12–2.95)	2.26 (2.08–2.61)	2.86 (2.36–3.10)	**<0.001**
SPINA-GT, pmol/s	2.16 (1.47–3.64)	2.67 (1.98–3.85)	1.47 (1.09–2.28)	**<0.001**
Anti-TPO positivity, *n* (%)	18 (18.9)	5 (8.9)	13 (33.3)	**0.003**
Anti-Tg positivity, *n* (%)	12 (12.6)	3 (5.4)	9 (23.1)	**0.011**
Presence of ≥1 positive thyroid autoantibody †, *n* (%)	21 (22.1)	8 (14.3)	13 (33.3)	**0.028**
Preoperative cytology				
Bethesda category, *n* (%)				0.941
Non-diagnostic (I)	4 (4.2)	2 (3.6)	2 (5.1)	
Benign (II)	14 (14.7)	9 (16.1)	5 (12.8)	
AUS/FLUS (III)	37 (38.9)	21 (37.5)	16 (41.0)	
Follicular neoplasm (IV)	8 (8.4)	4 (7.1)	4 (10.3)	
Suspicious for malignancy (V)	11 (11.6)	6 (10.7)	5 (12.8)	
Malignant (VI)	21 (22.1)	14 (25.0)	7 (17.9)	
Early postoperative ultrasonographic findings				
Remnant thyroid volume, mL	4.36 (3.07–6.38)	5.66 (3.44–7.01)	3.32 (2.35–4.60)	**<0.001**
Remnant thyroid volume-to-BSA ratio, mL/m^2^	2.47 (1.62–3.43)	2.74 (1.89–3.81)	1.79 (1.38–2.64)	**0.001**
Heterogeneous remnant thyroid parenchyma, *n* (%)	20 (21.1)	3 (5.4)	17 (43.6)	**<0.001**
Follow-up thyroid function				
Peak postoperative TSH during follow-up, mIU/L	3.71 (2.67–5.47)	2.86 (2.00–3.42)	6.06 (5.20–6.85)	**<0.001**
Lowest postoperative fT4 during follow-up, ng/dL	1.00 (0.94–1.10)	1.07 (0.98–1.16)	0.96 (0.90–1.02)	**<0.001**
Histopathological findings				
Malignant pathology, *n* (%)	54 (56.8)	30 (53.6)	24 (61.5)	0.441
Histopathological thyroiditis, *n* (%)	28 (29.5)	8 (14.3)	20 (51.3)	**<0.001**
Operated thyroid lobe (right), *n* (%)	47 (49.5)	29 (51.8)	18 (46.2)	0.589

Values are presented as median (interquartile range) for continuous variables and number (percentage) for categorical variables. Continuous variables were compared using the Mann–Whitney U test. Categorical variables were compared using Pearson’s χ^2^ test or Fisher’s exact test, as appropriate. Statistically significant *p* values are shown in bold. † The presence of a positive thyroid autoantibody was defined as positivity for anti-TPO and/or anti-Tg antibodies. **Abbreviations:** AUS/FLUS, atypia of undetermined significance/follicular lesion of undetermined significance; BMI, body mass index; BSA, body surface area; Anti-TPO, anti-thyroid peroxidase antibody; Anti-Tg, anti-thyroglobulin antibody; fT4, free thyroxine; SPINA-GT, thyroid secretory capacity (Structure Parameter Inference Approach); TSH, thyroid-stimulating hormone; TSHI, Thyroid Sensitivity Index (Jostel’s TSH Index).

**Table 2:** Univariable logistic regression analysis of predictors of postoperative hypothyroidism.

Variable	Cut-Off/Category	Odds Ratio (95% CI)	Nagelkerke R^2^	*p* Value
Preoperative TSH	≥1.77 mIU/L	5.63 (2.30–13.75)	0.205	<0.001
Preoperative TSHI	≥2.49	2.76 (1.18–6.43)	0.079	0.019
Preoperative SPINA-GT	≤2.10 pmol/s	6.36 (2.57–15.76)	0.230	<0.001
Remnant thyroid volume	≤4.24 mL	3.77 (1.59–8.92)	0.129	0.003
Remnant thyroid volume-to-BSA ratio	≤1.84 mL/m^2^	5.27 (2.14–13.00)	0.185	<0.001
Anti-TPO positivity	Positive	5.10 (1.64–15.86)	0.120	0.005
Anti-Tg positivity	Positive	5.30 (1.33–21.09)	0.090	0.018
Presence of ≥1 positive thyroid autoantibody †	Present	3.00 (1.10–8.17)	0.066	0.032
Heterogeneous remnant thyroid parenchyma	Present	13.65 (3.63–51.32)	0.267	<0.001

Continuous variables were dichotomized according to the optimal cut-off values determined by receiver operating characteristic (ROC) curve analysis using the Youden index. Nagelkerke R^2^ is reported as a measure of model explanatory performance. Odds ratios (ORs) and 95% confidence intervals (CIs) were derived from univariable binary logistic regression analyses. † The presence of ≥1 positive thyroid autoantibody was defined as positivity for anti-TPO and/or anti-Tg antibodies. **Abbreviations:** Anti-TPO, anti-thyroid peroxidase antibody; Anti-Tg, anti-thyroglobulin antibody; BSA, body surface area; CI, confidence interval; OR, odds ratio; ROC, receiver operating characteristic; SPINA-GT, thyroid secretory capacity; TSH, thyroid-stimulating hormone; TSHI, Jostel’s TSH index.

**Table 3:** Multivariable logistic regression models for prediction of postoperative hypothyroidism after hemithyroidectomy.

Variable	β	Adjusted OR (95% CI)	*p* Value	VIF
Model 1: Model with ultrasonographic parameters				
Preoperative TSH ≥ 1.77 mIU/L	1.622	5.06 (1.69–15.21)	0.004	1.078
Remnant thyroid volume-to-BSA ratio ≤ 1.84 mL/m^2^	1.890	6.62 (2.14–20.52)	0.001	1.054
Heterogeneous remnant thyroid parenchyma	3.036	20.83 (4.49–96.70)	<0.001	1.024
Model 2: Model without ultrasonographic parameters				
Preoperative TSH ≥ 1.77 mIU/L	1.815	6.14 (2.41–15.67)	<0.001	1.001
Presence of ≥1 positive thyroid autoantibody †	1.269	3.56 (1.17–10.85)	0.026	1.001

**Model performance:** Model 1 showed a model χ^2^ of 46.51 (*p* < 0.001), a Nagelkerke R^2^ of 0.522, a Hosmer–Lemeshow test *p* value of 0.768, and an overall classification accuracy of 80.0%. Model 2 showed a model χ^2^ of 20.93 (*p* < 0.001), a Nagelkerke R^2^ of 0.267, a Hosmer–Lemeshow test *p* value of 0.124, and an overall classification accuracy of 70.5%. **Model definitions:** Model 1 included preoperative TSH, remnant thyroid volume-to-BSA ratio, and heterogeneous remnant thyroid parenchyma. Model 2 was constructed without ultrasonographic parameters and included preoperative TSH and thyroid autoantibody positivity. VIF values were calculated to assess multicollinearity. † The presence of a positive thyroid autoantibody was defined as positivity for anti-thyroid peroxidase (anti-TPO) and/or anti-thyroglobulin (anti-Tg) antibodies. **Abbreviations:** BSA, body surface area; CI, confidence interval; OR, odds ratio; TSH, thyroid-stimulating hormone; VIF, variance inflation factor.

**Table 4:** Simplified clinical risk scores for predicting postoperative hypothyroidism after hemithyroidectomy.

Predictor	β Coefficient	Assigned Points
Model 1: Combined biochemical and ultrasonographic model		
Preoperative TSH ≥ 1.77 mIU/L	1.622	1
Remnant thyroid volume-to-BSA ratio ≤ 1.84 mL/m^2^	1.890	1
Heterogeneous remnant thyroid parenchyma	3.036	2
Model 2: Model without ultrasonographic parameters		
Preoperative TSH ≥ 1.77 mIU/L	1.815	2
Presence of ≥1 positive thyroid autoantibody	1.269	1
Total score	Risk category	Observed risk
Model 1		
0	Low	9.1%
1–2	Intermediate	44.7%
3–4	High	100%
Model 2		
0–1	Low	23.1%
2	Intermediate	51.5%
3	High	100%

Points were assigned according to the β regression coefficients derived separately from the corresponding multivariable logistic regression models and simplified while preserving the relative contribution of each predictor. Risk categories were defined according to the observed incidence of postoperative hypothyroidism in the study cohort. Model 1 had an AUC of 0.858 (95% CI, 0.779–0.936); at a cutoff of ≥2 points, sensitivity was 71.8% and specificity was 85.7%. Model 2 had an AUC of 0.745 (95% CI, 0.642–0.849); at a cutoff of ≥2 points, sensitivity was 69.2% and specificity was 71.4%. **Abbreviations:** AUC, area under the receiver operating characteristic curve; BSA, body surface area; CI, confidence interval; TSH, thyroid-stimulating hormone.

## Data Availability

The data presented in this study are available from the corresponding author upon reasonable request. The data are not publicly available because they contain information that could compromise the privacy of research participants.
